# Pallidal Deep Brain Stimulation for Tremor Control in a Child with Juvenile DNAJC6-Associated Parkinsonism

**DOI:** 10.5334/tohm.1187

**Published:** 2026-05-21

**Authors:** Katerina Bernardi, Deyana Valcheva, Rene Marquez Franco, Michael T. Barbe, Jochen Wirths, Anne Koy, Moritz Thiel

**Affiliations:** 1 Movement Disorders Program, Department of Neurology & F.M. Kirby Neurobiology Center, Boston Children’s Hospital, Harvard Medical School, Boston, MA 02115, USA; 2 Department of Pediatrics, Faculty of Medicine and University Hospital Cologne, University of Cologne, Cologne, 50937, Germany; 3 Department of Pediatrics, Pediatric Neurology, Sana Kliniken Duisburg, Germany; 4 Department of Stereotactic and Functional Neurosurgery, Faculty of Medicine and University Hospital Cologne, University of Cologne, Cologne, Germany; 5 Department of Neurology, Faculty of Medicine and University Hospital Cologne, University of Cologne, Cologne, Germany; 6 Center for Rare Diseases, Faculty of Medicine and University Hospital Cologne, University of Cologne, Cologne, 50937, Germany

**Keywords:** DNAJC6, Juvenile Parkinsonism, DBS, Tremor

## Abstract

**Background:**

Juvenile DNAJC6-associated Parkinson’s disease is a rare cause of early-onset parkinsonism with limited treatment data.

**Case Report:**

We describe a 15-year-old female with a novel DNAJC6 variant presenting with progressive tremor, bradykinesia, and postural instability. Although initially responsive to levodopa, she developed severe medication-induced motor fluctuations refractory to multiple pharmacological therapies. Due to disabling tremor, bilateral globus pallidus internus deep brain stimulation (GPi-DBS) was performed, resulting in marked and sustained tremor reduction.

**Discussion:**

This case highlights the severe course of DNAJC6-associated juvenile parkinsonism and supports GPi-DBS as an effective option for refractory tremor.

## Introduction

Juvenile DNAJC6-associated Parkinson’s disease is a rare autosomal recessive, early-onset neurodegenerative disorder characterized by parkinsonian features including bradykinesia, resting tremor, rigidity, and postural instability [1]. In addition, affected individuals frequently exhibit developmental delay, intellectual disability, epilepsy, dystonia, and neuropsychiatric symptoms, which may precede the onset of parkinsonism [2]. The disease course is often rapidly progressive and commonly associated with a poor or absent response to pharmacological treatment [3].

The DNAJC6 gene encodes a brain-specific isoform of auxilin, a protein that plays a critical role in clathrin-mediated synaptic vesicle recycling [4].

In this report, we describe a child with juvenile parkinsonism caused by a novel DNAJC6 variant who underwent bilateral globus pallidus internus deep brain stimulation (GPi-DBS), resulting in marked tremor improvement.

## Case Report

We present a 15-year-old female, born to healthy, non-consanguineous parents with no family history of neurological disorders. Pre- and perinatal history was unremarkable. During infancy, she exhibited muscular hypotonia and a significant speech delay. She did not crawl but achieved independent walking at 13 months and subsequently reached all further motor milestones. At 3.5 years of age, she was first diagnosed with autism spectrum disorder, although this diagnosis was not confirmed later.

At 10 years of age, she developed an irregular, medium-frequency, medium-amplitude tremor affecting the right arm and leg, accompanied by painful muscular spasms of the arms that improved with gabapentin. Over time, she experienced increasing difficulties with chewing and swallowing, excessive drooling, delayed bowel and bladder emptying and progressive speech impairment. A vertical gaze palsy emerged, followed by worsening bradykinesia, hypomimia and postural instability.

Metabolic investigations revealed mildly reduced cerebrospinal fluid dopamine metabolites (homovanillic acid and 5-hydroxyindoleacetic acid). EEG and brain MRI were unremarkable. She was clinically diagnosed with juvenile parkinsonism. Chromosomal microarray analysis identified a deletion at 16q24.1, and whole-exome sequencing revealed a novel homozygous splice-site variant in *DNAJC6* (c.543+1G>A), confirming the diagnosis at age 14 years.

This variant affects a canonical donor splice site and is predicted to disrupt normal splicing, resulting in aberrant transcript processing. Given that loss of function is a known disease mechanism for DNAJC6 [2], this variant constitutes very strong evidence of pathogenicity (PVS1) according to ACMG criteria [5] and has not been previously reported.

Over time tremor worsened, increasing in frequency and amplitude and presenting whole body distribution. Unfortunately, formal neurophysiological characterization of the tremor was not performed.

Levodopa–carbidopa therapy was initiated, but the patient exhibited marked drug sensitivity. Although levodopa effectively controlled the tremor, its benefit was short-lived: as soon as the drug effect wore off, tremor promptly re-emerged. Critically, any attempt to increase the dose to prolong or stabilize this response immediately triggered severe levodopa-induced dyskinesias (LID). In addition and particularly at night, prolonged compulsive pacing occurred with higher dosages of levodopa as possible manifestation of akathisia. Despite multiple treatment adjustments, including off-label administration of levodopa/carbidopa via gastrointestinal pump (4.7 mg/kg/day), the LID became intolerable. Dopamine agonists (oral and transdermal) and amantadine were ineffective, while trihexyphenidyl provided only transient benefit. Nitrazepam and dronabinol were used to improve sleep and relieve muscle cramps, and clonazepam was administered during severe tremor exacerbations, with transient benefit.

The alternating hyperkinetic and hypokinetic states became the dominant clinical feature, reflecting severe motor fluctuations and substantial therapeutic challenges (Video 1). The patient also developed recurrent vomiting and significant weight loss, necessitating gastrostomy followed by jejunostomy. Gastroscopy revealed severe gastroparesis.

**Video 1:** Patient’s clinical presentation before GPi-DBS, demonstrating cyclical alternation between hyperkinetic and hypokinetic states, reflecting severe motor fluctuations in relation to levodopa ON and OFF phases.

Due to progressive clinical deterioration and refractory motor fluctuations, bilateral GPi-DBS was performed at 14 years of age. Surgery was carried out under general anesthesia in a single stage, using a frame-based approach with intraoperative microelectrode recordings, and stimulation was activated immediately postoperatively. A dramatic and sustained reduction in tremor was observed, persisting throughout the entire follow-up period. LID initially resolved and independent ambulation was transiently restored but did not persist beyond the first months postoperatively; pharmacological therapy could nonetheless be reduced (Video 2).

**Video 2:** Patient’s clinical presentation after GPi-DBS, with active stimulation and on concurrent levodopa medication.

**Figure 1:**
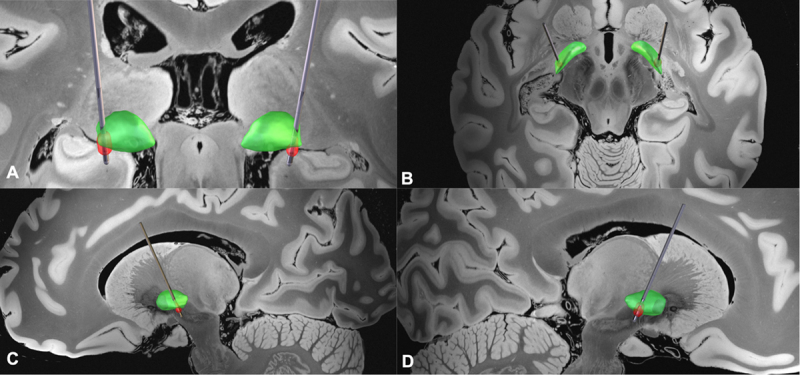
Visualization of electrode placement in a patient with DNAJC6-related juvenile Parkinsonism using Lead-DBS v3. The internal globus pallidus (GPi) is highlighted in green, with the Volume of Tissue Activated (VTA) represented in red. For spatial reference, the background features the 7-Tesla ex vivo human brain template. The patient was implanted with a Medtronic Sensight B33015 lead, and bilateral stimulation was applied with the following parameters: C (+); 1a,b,c (–) 30%, 2a,b,c (–) 70%; 2.2 mA, 60 µs, 130 Hz. Panel **(A)** presents a coronal view, **(B)** an axial view from above, **(C)** a sagittal left view, and **(D)** a sagittal right view.

Electrode localization is shown using Lead-DBS v3 visualization (Figure 1) [6,7]. Three months postoperatively, improvements were noted in Unified Parkinson’s Disease Rating Scale (UPDRS) and Caregiver Priorities and Child Health Index of Life with Disabilities (CPCHILD) scores (assessed under ongoing medication and active stimulation; Table 1).

**Table 1:** Clinical Scores.


	PRE GPI-DBS	3 MONTHS POST GPI-DBS	12 MONTHS POST GPI-DBS

UPDRS	177/260 Part I: 8 Part II: 52 Part III: 99 Part IV: 18	103/260 Part I: 7 Part II: 48 Part III: 48 Part IV: 0	Not assessed

CPCHILD	45/100	52/100	24/100


* UPDRS: Unified Parkinson’s Disease rating scale; CPCHILD: Caregiver Priorities and Child Health Index of Life with Disabilities. Table 1 presents the patient’s UPDRS and CPCHILD scores before and 3 months after GPi-DBS. CPCHILD was also repeated at 12 months post GPi-DBS. The scores were obtained during ongoing medical treatment and for post-DBS scores with ongoing stimulation. UPDRS assessment was not repeated at 12 months due to the severity of the patient’s neurological impairment at that time point; sustained tremor suppression at this stage reflects clinical observation and caregiver report. Lower scores on the UPDRS scale indicate better outcomes (i.e., less impairment or burden). Conversely, for the CPCHILD higher scores represent better outcomes in quality of life.

Despite sustained tremor control, the patient’s overall condition progressively worsened. She developed severe, painful dystonia and worsening postural instability refractory to stimulation adjustments. Once stimulation was briefly turned off and led to a severe increase of dystonia, making it unlikely a stimulation induced effect but more likely a symptom of disease progression. Moreover, clear capsular effects following stimulation were noticed while testing with higher amplitude and lowest contacts. Therefore, only contacts 2–4 were used for continuous stimulation. Detailed description of all stimulation settings tried over time are shown in Table 2. Formal ON/OFF medication and ON/OFF stimulation assessments were not feasible given the severity of the patient’s condition, precluding objective quantification of the DBS effect in isolation. Nevertheless, the consistent and immediate recurrence of tremor upon reduction of stimulation parameters provides meaningful, if informal, evidence of a stimulation-dependent therapeutic effect. Progressive neuropsychiatric symptoms emerged, including pronounced abulia, reduced speech output, and emotional lability.

**Table 2:** Evolution of Deep Brain Stimulation Parameters Over Time.


TIME AFTER GPI IMPLANTATION (MONTHS)	TARGET	CONTACTS/POLARITY	AMPLITUDE	PULSE WIDTH (µS)	FREQUENCY (HZ)	NOTES

0	GPi	–	–	–	–	DBS implantation (GPi)

0.0	GPi	Case (+), contacts 2–4 (–), 100% distribution	1.0 mA	60	130	Initial postoperative setting

0.4	GPi	Case (+), contacts 2–4 (–)	2.7 mA	60	130	Increase in amplitude

1.5	GPi	Case (+), contacts 2–4 (–)	3.1 mA	60	130	Increase in amplitude

3.4	GPi	Case (+), contacts 2–4 (–), 100% distribution	2.7 mA	90	130	Pulse width increased

10.8	GPi	Case (+), contacts 2–7 (–)	2.4 mA	90	130	Extensive testing, including low frequencies and low pulse widths without improvements; Contacts changed

13.6	STN	–	–	–	–	Additional DBS implantation (STN)

13.6	GPi	Case (+), contacts 2a–c / 10a–c (–)	2.4 mA	90	130	Not changed

13.6	STN	Case (+), L: 2a–c (–); R: 10a–c (–)	0.6 mA	60	130	Initial STN testing monopolar

13.9	STN	L: 2a–c (–), 3 (+); R: 10a–c (–), 3 (+)	0.3 mA	60	130	Bipolar stimulation

14.0	GPi	Case (+), contacts 2a–c / 10a–c (–)	2.4 mA	90	130	Not changed

14.0	STN	Case (+), L: 2a–c (–); R: 10a–c (–)	0.8 mA	60	130	Increased STN amplitude

14.2	GPi	Case (+), contacts 2a–c / 10a–c (–)	2.4 mA	90	130	Not changed

14.2	STN	Case (+), L: 2a–c (–); R: 10a–c (–)	1.2 mA	60	130	Increased STN amplitude


Shown are the sequential DBS programming settings after initial GPi implantation and later additional STN implantation. For each timepoint, the target, contact configuration and polarity, amplitude, pulse width, frequency, and relevant programming notes are provided. GPi stimulation was initially adjusted by stepwise increases in amplitude and pulse width. Because extensive GPi programming modifications did not result in meaningful improvement, supplementary STN stimulation was introduced and gradually escalated, including testing of both monopolar and bipolar configurations, whereas GPi stimulation parameters were maintained.

Continuous subcutaneous foslevodopa/foscarbidopa infusion (8–10 mg/kg/day) was initiated to avoid LID and gastrointestinal intolerance of oral treatment but yielded only transient benefit. Dyskinesias recurred even at low doses, accompanied by akathisia, leading to frequent falls and injuries. Pump therapy was discontinued, and clonidine with chloral hydrate (up to 0.6 µg/kg/hour) was introduced at night, resulting in modest improvement of dystonia and sleep.

Approximately one year after GPi-DBS, despite persistent tremor suppression, the patient exhibited severe disease progression with intractable dystonia and paroxysmal exacerbations, necessitating transfer to palliative care, a trajectory reflected in the 12-month CPCHILD scores (Table 1). Subsequent bilateral subthalamic nucleus (STN) DBS implantation did not result in clinical improvement.

## Discussion

This case highlights the complexity of clinical and pharmacological management in juvenile *DNAJC6*-associated parkinsonism. Multiple phenotypes have been described, including parkinsonism-predominant [4], dystonia-predominant [8], dystonia–parkinsonism [3,9], and generalized dystonia with tremor-dominant parkinsonism [10]. While many reported patients initially respond to levodopa, motor complications, dyskinesias, and behavioral symptoms often severely impair quality of life [9,11].

Our patient harbored a novel DNAJC6 variant and exhibited a rapidly progressive, parkinsonism-predominant phenotype with severe tremor and profound levodopa intolerance.

Given the rapidly worsening clinical condition and the unsatisfactory pharmacological treatment, GPi-DBS was performed. Due to the significant dyskinesia observed in our patient, GPi-DBS was preferred over STN-DBS, as it may provide superior control of hyperkinetic symptoms [12].

To date, DBS experience in *DNAJC6*-associated disease remains extremely limited. Previously reported adult patients with early-onset *DNAJC6*-related parkinsonism showed good levodopa responsiveness but developed severe motor complications; surgical interventions included pallidotomy or STN-DBS with favorable outcomes [11,13].

A recent report describes a 19-year-old female carrying a novel DNAJC6 mutation who presented predominantly with dystonia and showed only a poor response to pharmacological treatment, prompting bilateral GPi-DBS implantation [14]. In that case, GPi-DBS resulted in a marked and sustained improvement of dystonia as well as tremor, allowing for a substantial reduction of dopaminergic medication and leading to an overall functional benefit.

In contrast, the clinical presentation of our patient differed considerably. Parkinsonian features with severe, functionally disabling tremor initially predominated over dystonia and were accompanied by pronounced LID and extreme motor fluctuations. The most clinically meaningful effect of GPi-DBS in our case was a dramatic and sustained suppression of tremor, which persisted throughout the entire follow-up period and proved resistant to stimulation adjustments. This pronounced anti-tremor effect represents a relevant therapeutic achievement in DNAJC6-associated juvenile parkinsonism, a condition in which effective treatment options are exceedingly limited.

However, despite the robust tremor control, beneficial effects on other motor and non-motor symptoms—including LID, sleep disturbances, independent ambulation, and medication burden—were only transient and failed to translate into a sustained improvement in quality of life. Importantly, in contrast to the previously reported dystonia-predominant case [14], our patient did not experience meaningful or lasting improvement of dystonia following GPi-DBS. Dystonia subsequently progressed to become the dominant and most treatment-refractory feature, ultimately driving overall disease progression. Given this unfavorable course, additional bilateral STN-DBS was attempted; however, this intervention did not result in any clinical benefit.

This trajectory raises important considerations regarding surgical risk-benefit assessment in rapidly progressive dystonia-parkinsonism syndromes, where DBS may offer only palliative rather than disease-modifying benefit and procedural risks must be carefully weighed against uncertain therapeutic gain. In DNAJC6-associated parkinsonism, published DBS experience remains extremely limited and outcomes appear heterogeneous and phenotype-dependent. While refractory tremor would likely have further compromised our patient’s quality of life in the absence of intervention, the overall disease trajectory was not altered, underscoring the importance of transparent shared decision-making with patients and families before proceeding with surgery.

In conclusion, this case demonstrates that *DNAJC6*-associated juvenile parkinsonism can follow a devastating course refractory to standard medical therapies. While GPi-DBS may provide effective and sustained tremor control, it does not modify overall disease progression and should be regarded as a palliative intervention. Nevertheless, given the scarcity of published data on DBS outcomes in juvenile *DNAJC6*-related parkinsonism, our experience suggests that GPi-DBS can be considered a viable therapeutic option specifically for tremor control, while emphasizing the need for realistic prognostic counseling regarding overall disease progression.
